# Extramedullary plasmacytoma of the ureter in an HIV-positive patient

**DOI:** 10.1007/s13691-017-0300-3

**Published:** 2017-05-27

**Authors:** Takashi Nagai, Takehiko Okamura, Yosuke Taki, Yutaro Tanaka, Daichi Kobayashi, Takahiro Kobayashi, Hidetoshi Akita, Takahiro Yasui

**Affiliations:** 1 Department of Urology, Anjo Kosei Hospital, 28 Higashihirokute, Anjo-cho, Anjo, Aichi-ken 446-8602 Japan; 2 Department of Nephro-Urology, Nagoya City University, Graduate School of Medical Sciences, Nagoya, Japan

**Keywords:** Extramedullary plasmacytoma, Ureter, HIV infection

## Abstract

A 45-year-old Japanese man, who was undergoing HIV infection treatment, was aware that he had gross hematuria, and he was diagnosed as having a ureteral tumor by radiographic examination. Therefore, he was referred to our department for further examination and treatment. We considered that the ureteral tumor was a urothelial carcinoma (cT2N0M0) because of the left ureteral tumor and urine cytology results, and thus, laparoscopic ureteronephrectomy was performed. The pathological diagnosis was a solitary extramedullary plasmacytoma (EMP) of the ureter. Currently, he is alive and free of disease at 7 months postoperatively. EMP develops in the nasal cavity, paranasal cavity, gastrointestinal tract, lung, thyroid, eye socket, lymph node, and various organs, but the ureter is an extremely rare site of EMP. In addition, the patient had an HIV infection. To the best of our knowledge, this is the first case of EMP of the ureter in an HIV-positive patient.

## Introduction

A plasmacytoma is a solitary mass of neoplastic monoclonal plasma cells in either bone or soft tissue. An extramedullary plasmacytoma (EMP) is less common than a solitary bone plasmacytoma, and it occurs when there is soft tissue infiltration of clonal plasma cells [1]. An EMP occurs in the nasal cavity, paranasal cavity, gastrointestinal tract, lung, thyroid, eye socket, lymph node, and various organs [1], but the ureter is an extremely rare site of EMP. We describe a patient with an EMP in the ureter who had an HIV infection. To the best of our knowledge, this is the first case of an EMP of the ureter in an HIV-positive patient.

## Case report

**Fig. 1:**
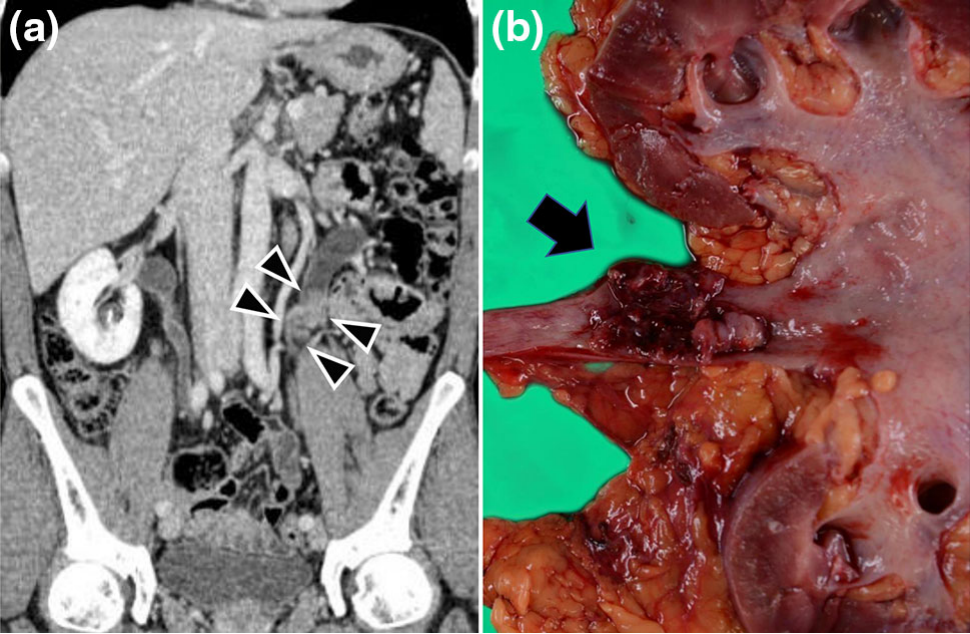
**a** Enhanced computed tomography image in the coronal view. A mass (*arrowhead*) causing hydroureter is noted in the *left* ureter. **b** Macroscopic finding of the resected specimen. A solid, papillary tumor measuring 30 mm in diameter (*arrow*) is observed in the *upper side* of the ureter

**Fig. 2:**
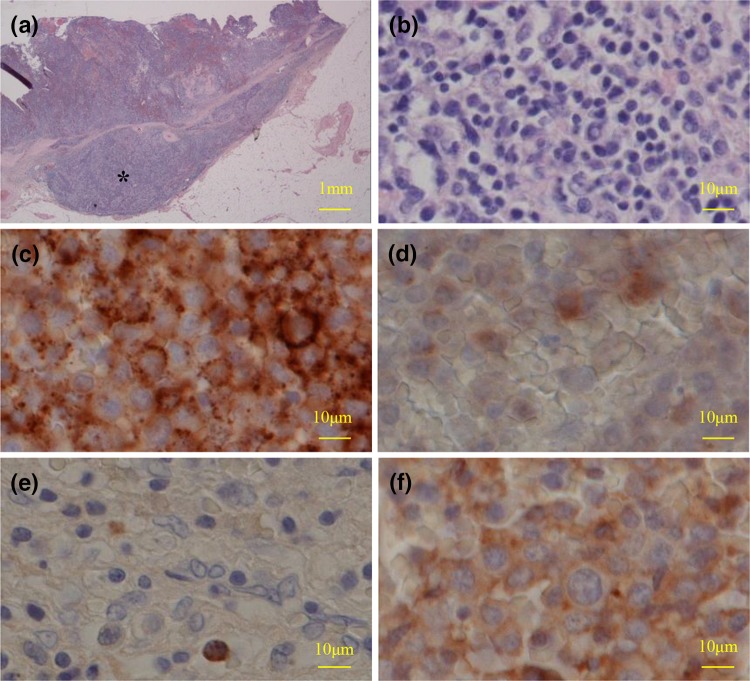
**a** In the low-power field, tumors (*asterisk*) invade at least the periureteral soft tissue (hematoxylin and eosin staining). **b** In the high-power field, tumor cells are plasmacytoma-like atypical cells with a high nuclear-cytoplasmic ratio (hematoxylin and eosin staining). **c** In the high-power fields, the tumor cells are positive for CD138. **d** In the high-power fields, the tumor cells are positive for CD79a/mb-1. **e** In the high-power field, tumor cells are positive for immunoglobulin (Ig)κ. **f** In the high-power field, tumor cells are positive for Igλ. Since staining is stronger for Igλ than for Igκ, light chain restriction (Igλ > Igκ) is observed

A 45-year-old Japanese man receiving HIV infection treatment had gross hematuria. Since the ultrasound sonogram and computed tomography (CT) scan showed a left ureteral tumor (Fig. 1a), he was presented to our department for further examination and treatment. His medical history included condylomata acuminate of the penis, hepatitis B, and HIV infection. The HIV infection was well controlled with dolutegravir and emtricitabine/tenofovir. He had no significant family, allergic, or smoking history. He received no blood transfusions. Serum laboratory findings showed an increased creatinine level (1.23 mg/dL; normal range <1.2 mg/dL) but no increase in tumor markers such as the squamous cell carcinoma antigen and cancer antigen 19-9. Results of urinalysis showed hematopyuria, and urine cytology findings were pseudo-positive for urothelial carcinoma, of which few cells had a high nuclear-cytoplasmic ratio and their nuclei were hyperchromatic. On cystoscopy, no bladder tumor was observed. A whole-body CT scan was performed, and no distant metastasis or lymph node involvement was found. We also conducted retrograde unilateral left pyelography and a selective upper urinary cytology examination. The pyelogram showed the same left ureteral mass that was found on the CT scan. Results of the left upper urinary cytology examination were negative; there were few atypical cells, but their nuclei were not hyperchromatic. We diagnosed the ureteral tumor as a urothelial carcinoma (cT2N0M0) because of the left ureteral tumor and abnormal urine cytology findings, and laparoscopic ureteronephrectomy was performed. Macroscopically, a solid, papillary tumor, 30 mm in diameter, was observed in the upper side of the ureter (Fig. 1b). Histopathological findings included plasmacytoma-like atypical cells with a high nuclear-cytoplasmic ratio, perinuclear halo formation of invasion at the periureteral soft tissue of the ureter (Fig. 2a, b), and lymphocytic and histiocytic infiltration in the tumor. Invasion of the tumor cells was not observed in the pelvic mucosa or renal parenchyma. There was no component of urothelial carcinoma in the tumor or surrounding mucosa of the ureter. Immunohistochemical staining was performed to confirm the definitive diagnosis. Tumor cells were negative for cytokeratin (CK) 7, CK20, and p63 but positive for the markers of B cells or plasma cells, such as CD138 and CD79a/mb-1 (Fig. 2c). In addition, light chain restriction [immunoglobulin (Ig)γ > Igκ] was observed (Fig. 2d). The final histopathological diagnosis was plasmacytoma not urothelial carcinoma.

Postoperatively, the patient recovered without any complications. It was necessary to explore the entire body to find any other plasmacytomas. The positron emission tomography scan showed no other lesions. Bone marrow aspiration findings were normal, and radiographs of the skull, spine, limbs, and pelvis showed no osteolytic lesions. High levels of monoclonal protein were not found in the blood or urine. Finally, the definitive diagnosis was solitary extramedullary plasmacytoma of the ureter (Durie and Salmon criteria, stage 1). He is alive and free of disease at 7 months postoperatively.

## Discussion

The main sites of EMP are the upper respiratory tract and upper gastrointestinal tract, both accounting for approximately 80% of cases. The ureter is an extremely rare site of EMP, and only 3 cases, including the present case, have been reported in the literature (Table 1) [2, 3].

**Table 1:** Clinical features of reported cases of EMP in ureter

Case no.	Authors	Year	Age	Sex	Chief complaint	Treatment	Follow-up (months)	Outcome
1	Landsmann S	2009	80	Female	Renal colic	Segmental resection and ureterocystoneostomy	–	No evidence of disease
2	Klein T	2010	82	Female	Hematuria	Nephroureterectomy	–	No evidence of disease
3	Nagai T	2016	45	Male	Hematuria	Nephroureterectomy	5	No evidence of disease

*EMP* extramedullary plasmacytoma

The International Myeloma Working Group defined EMP by the following criteria: (1) no monoclonal Ig in serum or urine; (2) a tumor composed of monoclonal plasma cells in a single extramedullary site; (3) no lesion in the bone marrow; (4) no lesion in the whole-body bone; and (5) no involvement of organs [4]. Diagnosing EMP of the ureter preoperatively may be difficult. The clinical features of EMP were completely different among three patients, including our case. Radiographic images such as CT or magnetic resonance imaging may be useful, but a ureteral mass is nonspecific. Bone marrow biopsies and fine needle aspirations as well as urine and blood tests are obligatory [3]. However, there is no standard treatment for EMP of the ureter. Surgical excision, radiotherapy, chemotherapy, or combined surgery and radiotherapy are considered. Landsmann et al. [2] reported that they conducted segmental removal of the affected ureter and ureterocystostomy, but Klein et al. [3] reported that nephroureterectomy was performed because of a suspected diagnosis of a transitional cell carcinoma of the ureter. We performed nephroureterectomy in our case, because the ureteral mass on the CT scan was definitive for urothelial carcinoma and urine cytology findings were pseudo-positive. Although it is difficult to diagnose plasmacytomas preoperatively, avoiding nephrectomy may be considered based on ureteroscopy findings and biopsy results in patients with acquired solitary kidney or renal function degeneracy. In general, the National Comprehensive Cancer Network panel recommends radiotherapy as the primary treatment for the involved field followed by operation, if necessary, for an EMP [5]. In a patient with an undiagnosed ureteral tumor, ureteronephrectomy may be standard treatment considering the possibility of a malignant tumor such as urothelial carcinoma.

In this case, the patient had an HIV infection, but there were no atypical features in contrast to a ureteral tumor such as urothelial carcinoma. Adequate informed consent for the patient or family is needed, because the pathological results may be atypical, especially in patients with an HIV infection. In addition, an EMP tends to occur with HIV in a younger person and can be more aggressive because of poor immunity [6]. In view of a high incidence of progression to multiple myeloma in due course, patients should be kept under constant surveillance [6]. Though the relationship between a plasmacytoma and HIV infection is still unclear, it has been reported that multiple myeloma in HIV-infected patients shows an atypical clinical evolution; it tends to present as a solitary bone plasmacytoma or extramedullary plasmacytoma [7]. The pathogenesis of a plasmacytoma in patients with an HIV infection is unknown, but chronic antigen stimulation and immunodeficiency are believed to be the driving forces of the process [8].

## Conclusion

When treating HIV-positive patients, physicians should consider abnormal tumors such as a plasmacytoma when making a differential diagnosis.

## Abbreviations

EMP: Extramedullary plasmacytoma
CT: Computed tomography
CK: Cytokeratin
